# Supplementary material for: 2D perovskite stabilized phase-pure formamidinium perovskite solar cells
Source: Nat Commun. 2018 Aug 1;9:3021. doi: 10.1038/s41467-018-05454-4 (PMC6070510; doi:10.1038/s41467-018-05454-4)
Supplement: Supplementary file 1 — Supplementary Information [file 41467_2018_5454_MOESM1_ESM.pdf]

Supplementary information

2D perovskite stabilized phase-pure formamidinium perovskite solar cells

Jin-Wook Lee, Zhenghong Dai, Tae-Hee Han, Chungseok Choi, Sheng-Yung Chang, Sung-Joon Lee, Nicholas De Marco, Hongxiang Zhao, Pengyu Sun, Yu Huang and Yang Yang*

*Corresponding author

E-mail: yangy@ucla.edu

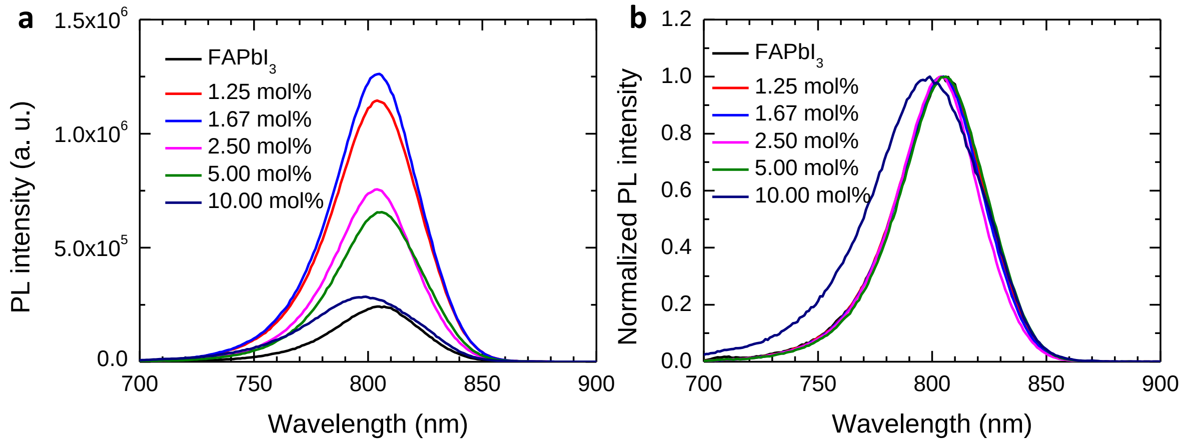

Supplementary Figure 1| Effect of 2D perovskite on photoluminescence properties. Steady-state photoluminescence spectra of $FAPbI_3$ perovskite film with different amount of added 2D PEA_2PbI_4 perovskite. (**a**) Original and (**b**) normalized spectra.

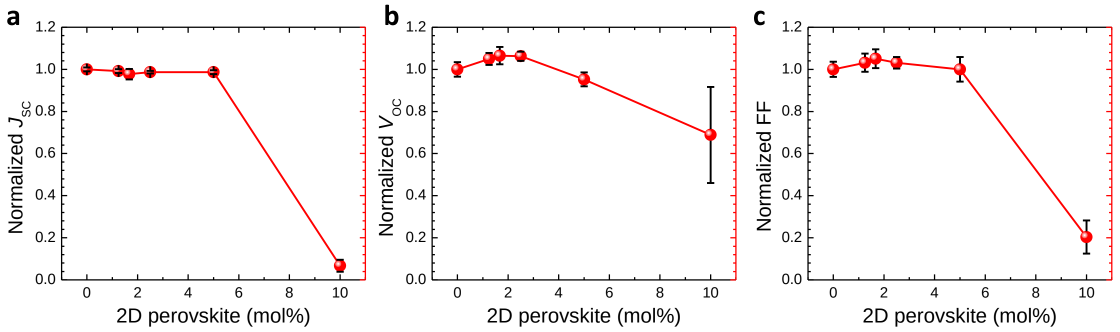

Supplementary Figure 2| Effect of 2D perovskite on photovoltaic performance. Normalized (**a**) short-circuit current density (J_{SC}), (**b**) open circuit voltage (V_{OC}) and (**c**) fill factor (FF) of planar $FAPbI_3$ perovskite solar cells with different amount of the added 2D PEA_2PbI_4 perovskite. The photovoltaic parameters are obtained from reverse scan (from V_{OC} to J_{SC}) with scan rate of 0.1 Vs^{-1}. The error bar of the normalized PCE indicates standard deviation of the PCEs. At least 10 devices were fabricated for each condition.

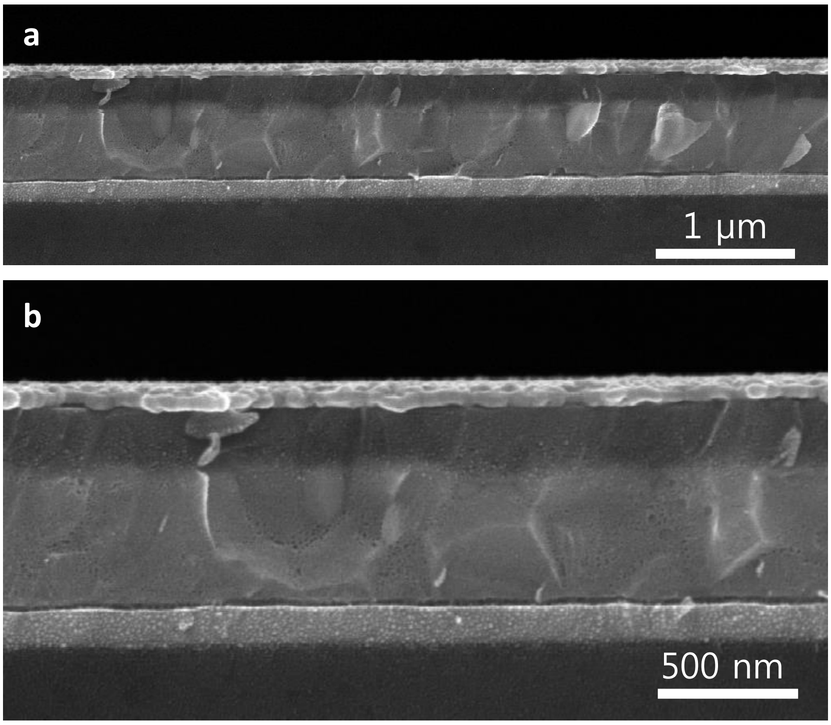

Supplementary Figure 3| Cross sectional scanning electron microscopic (SEM). Cross-sectional SEM images of the device with 1.67 mol% of PEA_2PbI_4 2D perovskite**.** The structure of the device is ITO/compact-SnO_2/perovskite/spiro-MeOTAD/Ag or Au. (**a**) Lower and (**b**) higher magnification images.

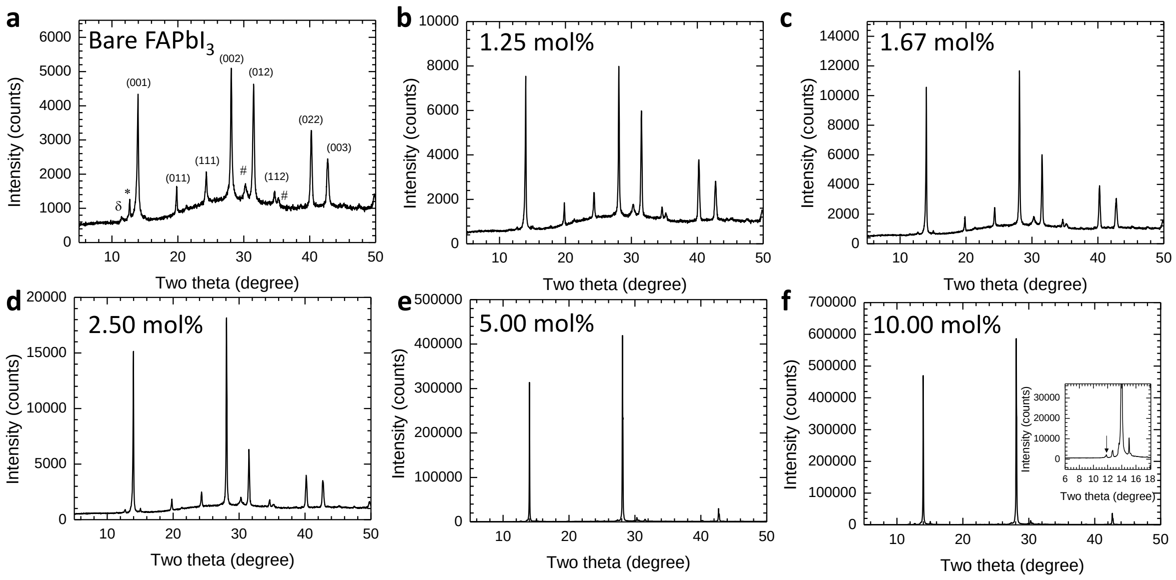

Supplementary Figure 4| X-ray diffraction patterns with different amount of 2D perovskite. (**a**) bare $FAPbI_3$, with (**b**) 1.25 mol%, (**c**) 1.67 mol%, (**d**) 2.50 mol%, (**e**) 5.00 mol% and (**f**) 10.00 mol% 2D PEA_2PbI_4 perovskite. Peaks from cubic $FAPbI_3$ phase were indexed in a. δ and * indicate hexagonal $FAPbI_3$ and PbI_2, respectively.

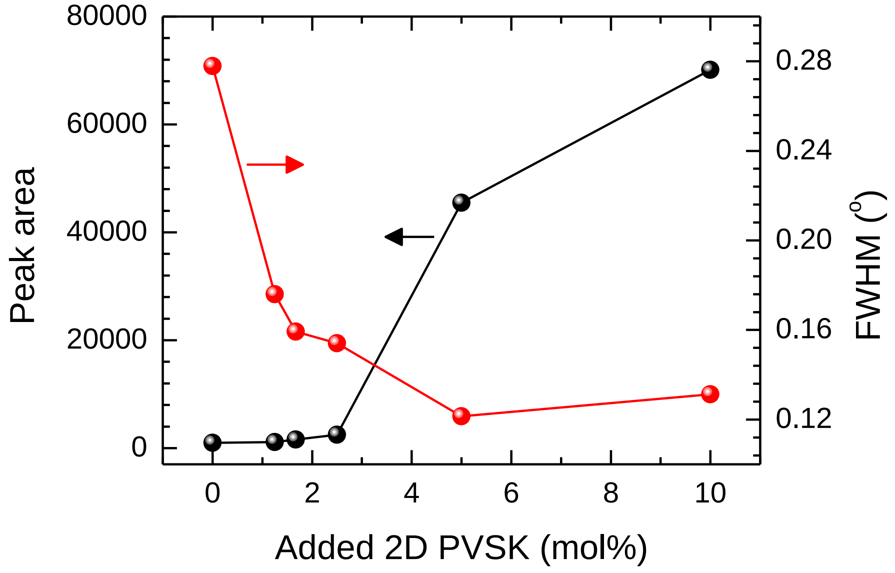

Supplementary Figure 5| Crystallinity dependent on added 2D perovskite. Peak area and full-width-half-maximum (FWHM) calculated from X-ray diffraction patterns with different amount of PEA_2PbI_4 in Supplementary Figure 4. The (002) peak (at around 28 ^{o}) was used for the Gauss fit.

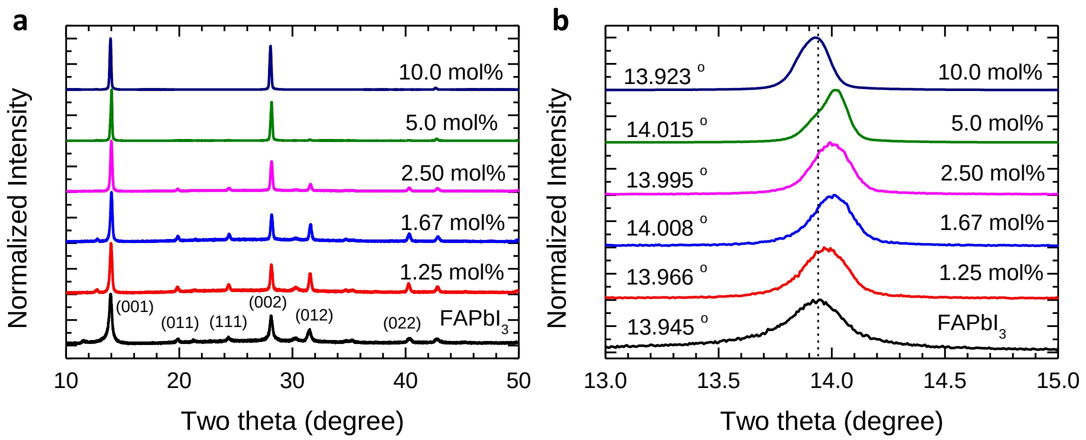

Supplementary Figure 6| Normalized X-ray diffraction (XRD) patterns. Normalized XRD patterns of $FAPbI_3$ perovskite films with different amount of added 2D PEA_2PbI_4 perovskite. (**a**) full spectra and (**b**) magnified (001) orientation peaks

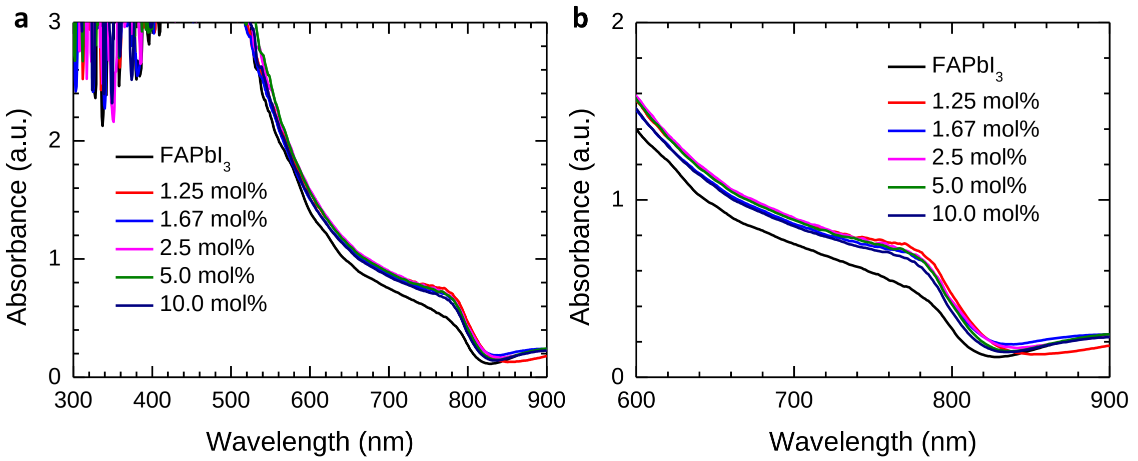

Supplementary Figure 7| Effect of added 2D perovskite on absorption spectra. Absorption spectra of $FAPbI_3$ perovskite films with different amount of added 2D PEA_2PbI_4 perovskite. (**a**) full spectra and (**b**) magnified onset region

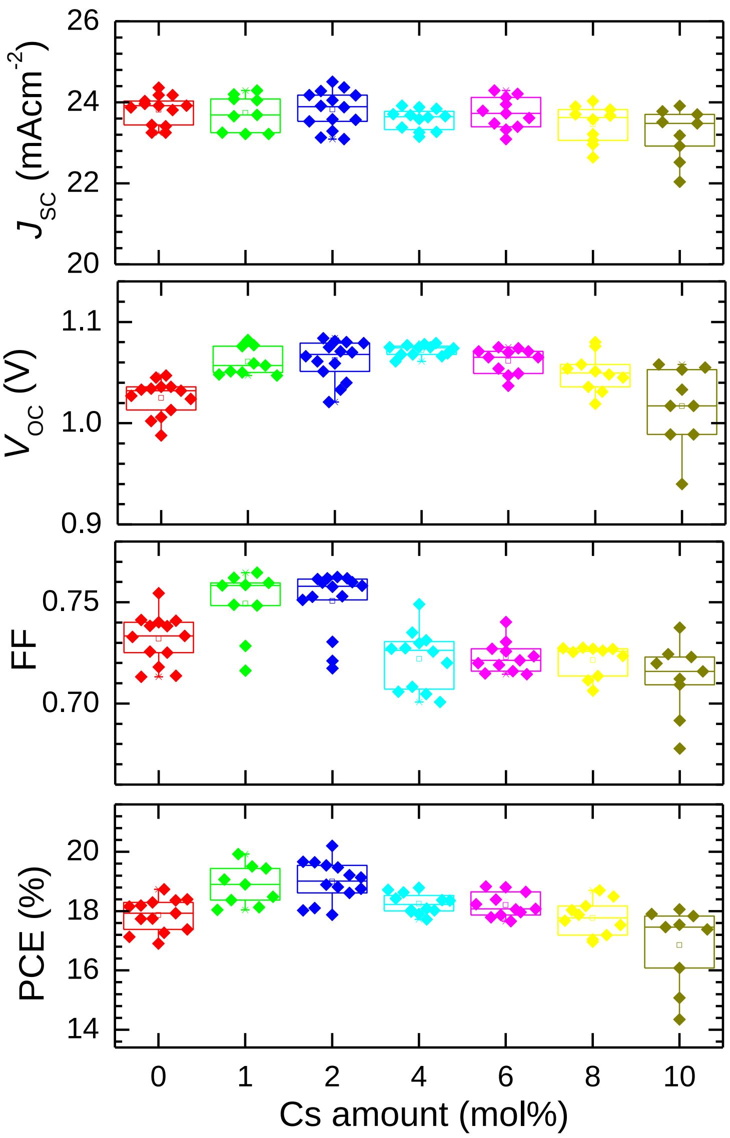

Supplementary Figure 8| Effect of cesium (Cs) amount on photovoltaic performance. Short-circuit current density (J_{SC}), open circuit voltage (V_{OC}), fill factor (FF) and power conversion efficiency (PCE) of $FAPbI_3$ perovskite solar cells incorporating 1.67 mol% PEA_2PbI_4 and different Cs amount. The photovoltaic parameters are obtained from reverse scan (from V_{OC} to J_{SC}) with scan rate of 0.1 Vs^{-1}.

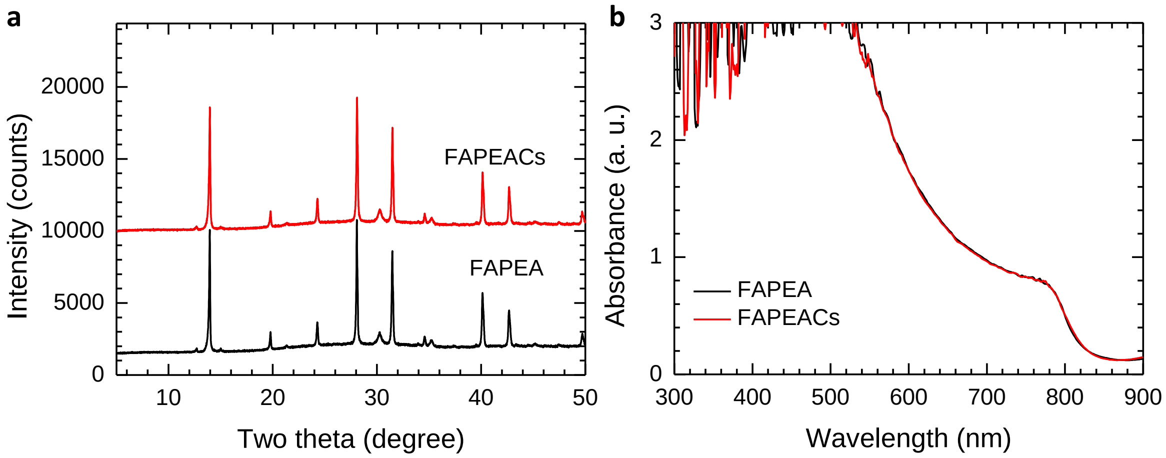

Supplementary Figure 9| Effect of Cs on crystal structure and absorption. Comparison of (**a**) X-ray diffraction pattern and (**b**) absorption spectra of $FAPbI_3$ with 1.67 mol% PEA_2PbI_4 (FAPEA) and $FA_{0.98}Cs_{0.02}PbI_3$ with 1.67 mol% PEA_2PbI_4 (FAPEACs).

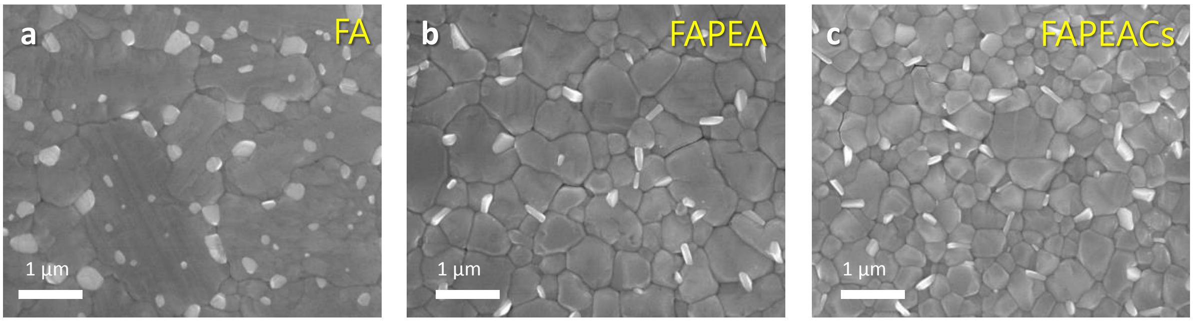

Supplementary Figure 10| Effect of Cs on morphology of perovskite film. Surface scanning electron microscopic (SEM) images of **a**, bare $FAPbI_3$ (FA), **b**, with 1.67 mol% PEA_2PbI_4 (FAPEA) and **c**, $FA_{0.98}Cs_{0.02}PbI_3$ with 1.67 mol% PEA_2PbI_4 (FAPEACs).

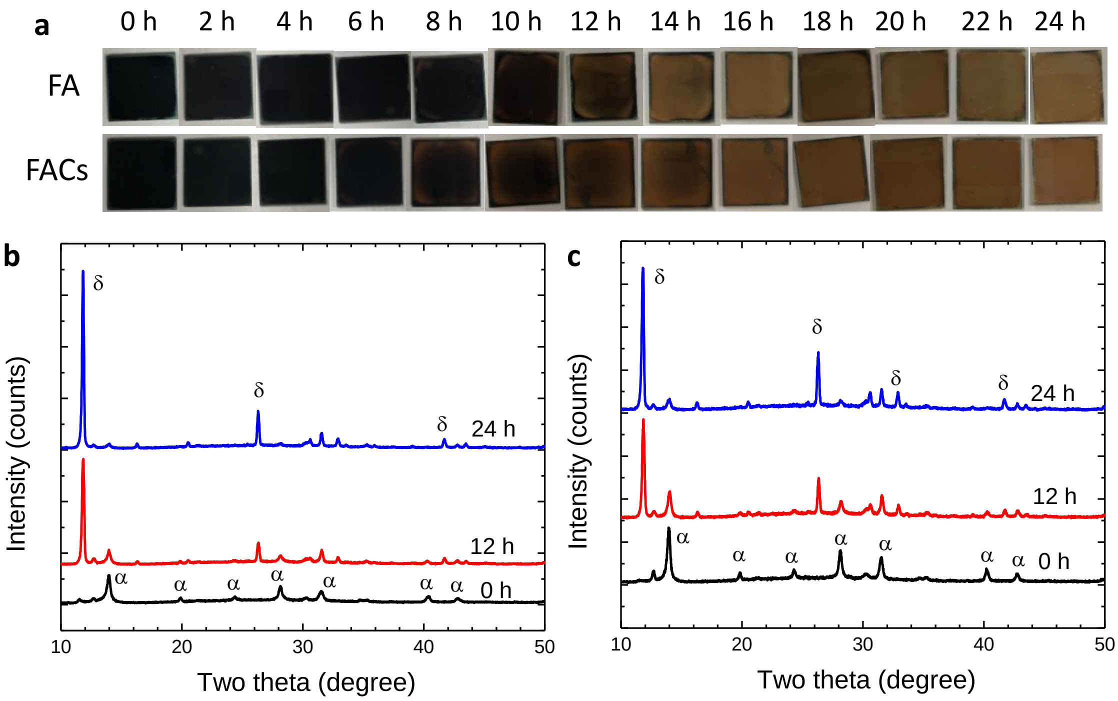

Supplementary Figure 11| Humidity stability of $FAPbI_3$ and $FA_{0.98}Cs_{0.02}PbI_3$ perovskites. (**a**) photos and (**b**, **c**) X-ray diffraction patterns of the $FAPbI_3$ and $FA_{0.98}Cs_{0.02}PbI_3$ perovskite films with exposure to relative humidity (RH) of 70±5% at 20±2 ^{o} for different time. Peaks from cubic $FAPbI_3$ phase were indexed by α whereas δ and * indicate hexagonal $FAPbI_3$ and PbI_2, respectively.

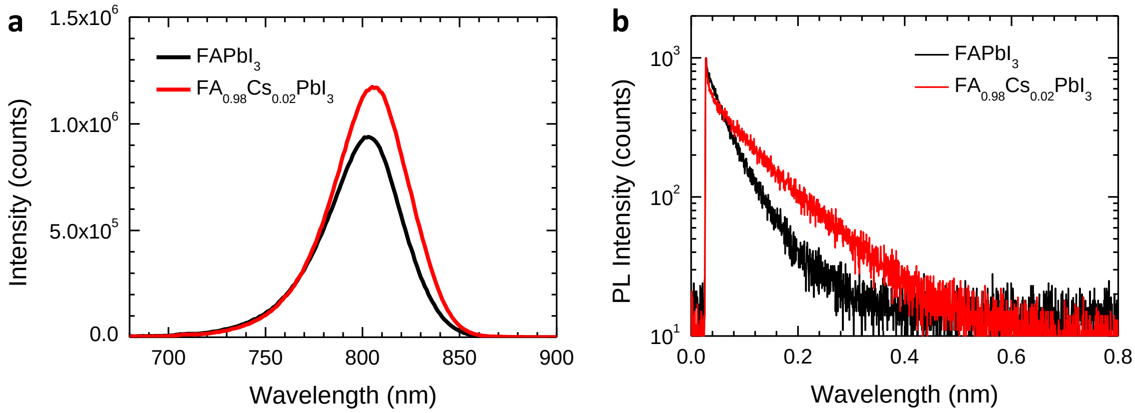

Supplementary Figure 12| Photoluminescence (PL) properties. (**a**) Steady-state and (**b**) time-resolved PL measurements of the $FAPbI_3$ and $FA_{0.98}Cs_{0.02}PbI_3$ perovskite films.

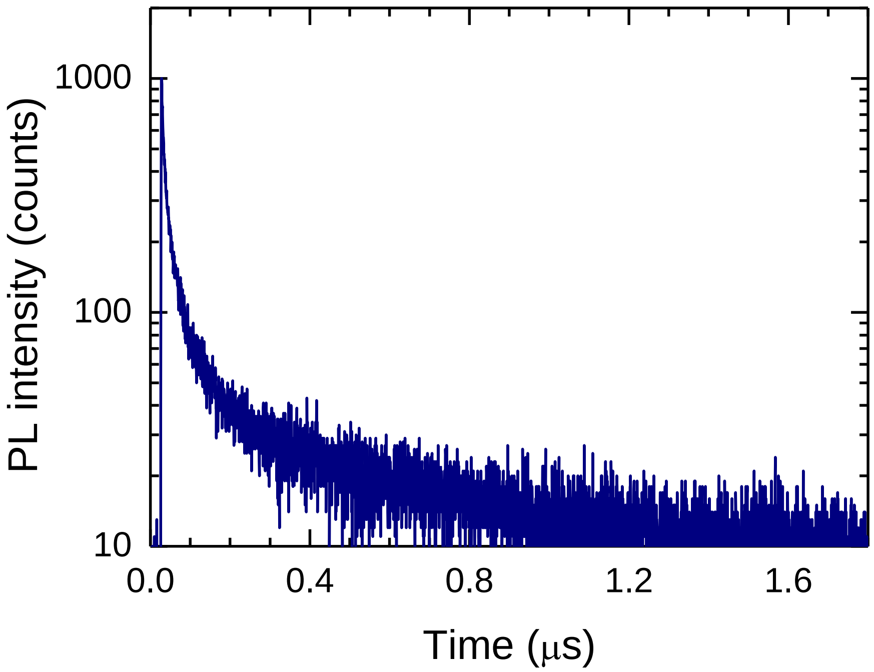

Supplementary Figure 13| Photoluminescence decay profile of quasi-3D perovskite. Time-resolved photoluminescence decay profile of the $FAPbI_3$ perovskite film with incorporation of 10 mol% of PEA_2PbI_4 perovskite.

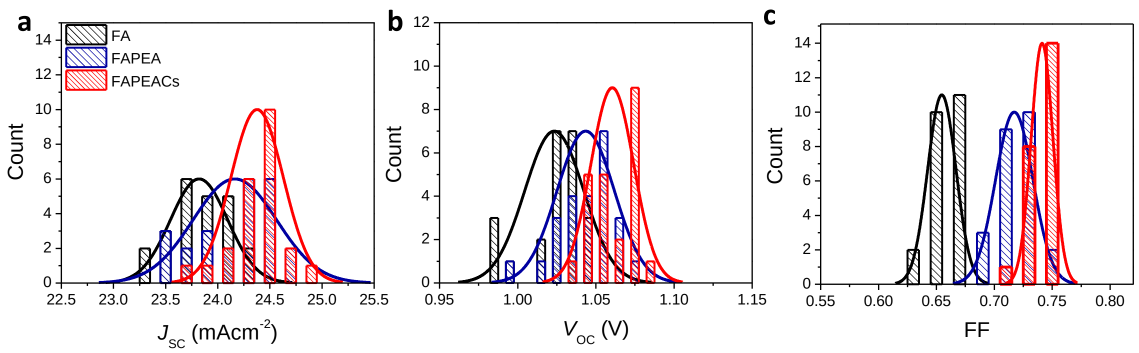

Supplementary Figure 14| Photovoltaic parameters with 2D perovskite and Cs. (**a**) Short-circuit current density (J_{SC}), (**b**) open circuit voltage (V_{OC}) and (**c**) fill factor (FF) of the device incorporating bare $FAPbI_3$ (FA), $FAPbI_3$ with 1.67 mol% PEA_2PbI_4 (FAPEA) and $FA_{0.98}Cs_{0.02}PbI_3$ with 1.67 mol% PEA_2PbI_4 (FAPEACs). The photovoltaic parameters are obtained from reverse scan (from V_{OC} to J_{SC}) with scan rate of 0.1 Vs^{-1}. All the devices were fabricated in same batch.

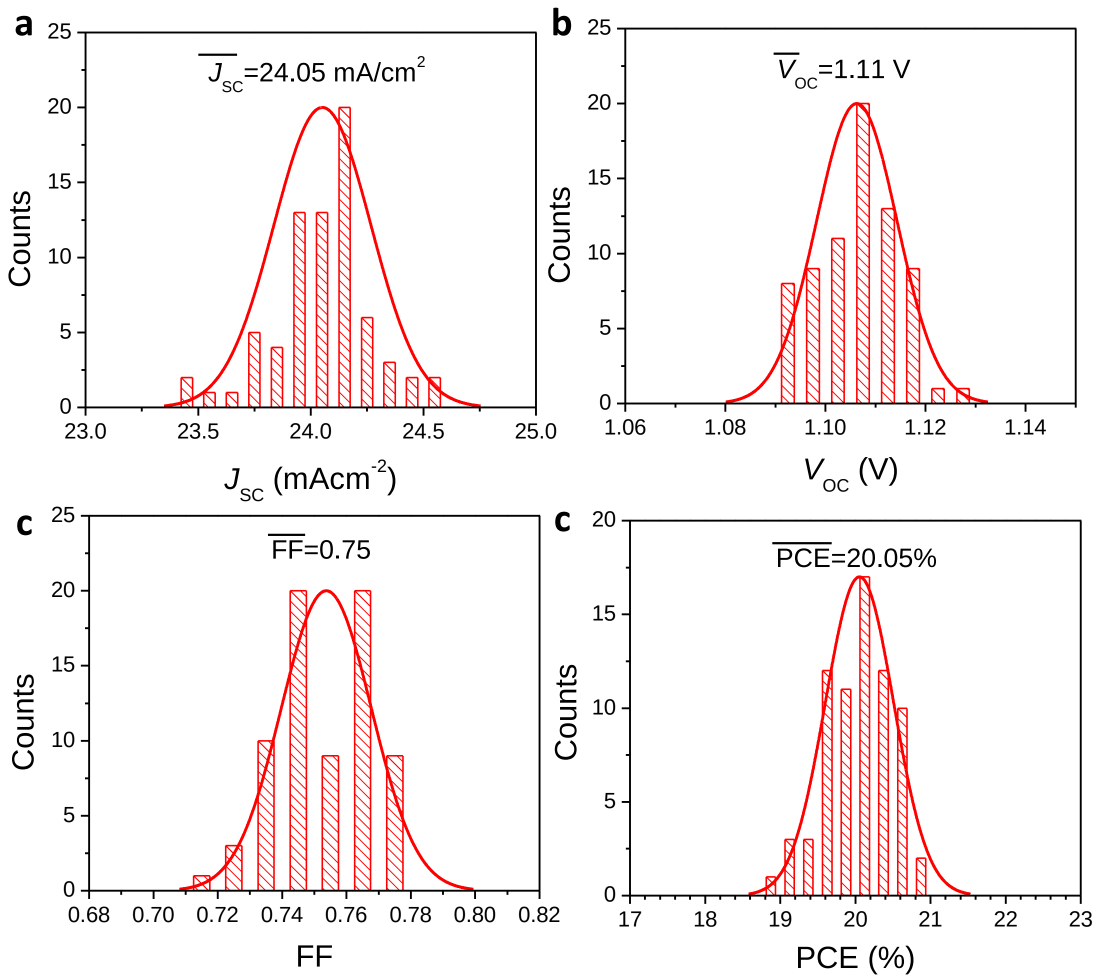

Supplementary Figure 15| Distribution of photovoltaic parameters for target devices. (**a**) Short-circuit current density (J_{SC}), (**b**) open circuit voltage (V_{OC}), (**c**) fill factor (FF) and (**d**) power conversion efficiency (PCE) of the device incorporating $FA_{0.98}Cs_{0.02}PbI_3$, with 1.67 mol% PEA_2PbI_4. 74 devices were fabricated from two different batches.

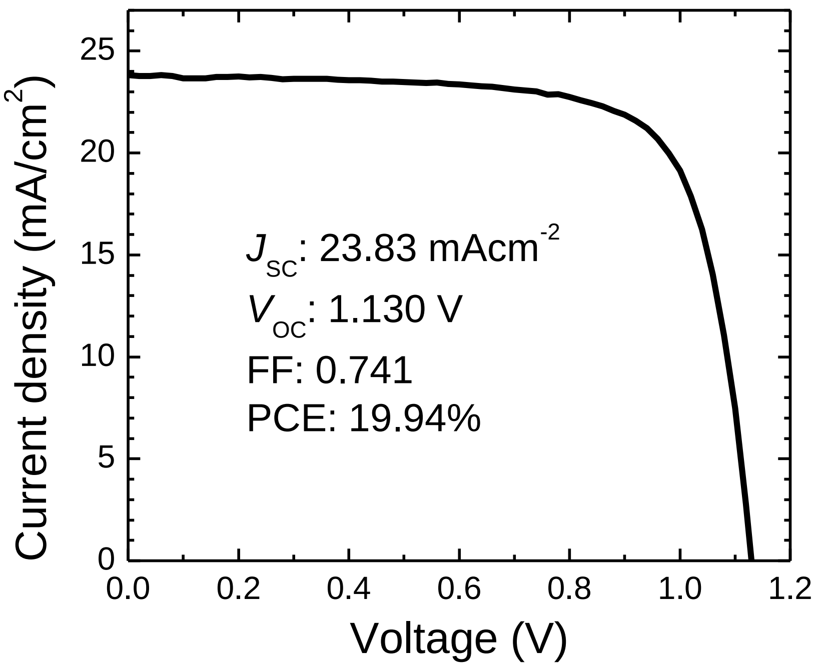

Supplementary Figure 16| A target device with the highest open-circuit voltage (V_{OC}). Current density-voltage (J-V) curve of the target device showing the highest V_{OC}. The *J-V* curve was obtained from reverse scan with scan rate of 0.1 Vs^{-1}.

Technology and Application Center

PV Lab

Newport Calibration Cert. # 1752

DUT S/N: 1752 D19
Newport Calibration #: 1752
Manufacturer: UCLA Yang Yang
Material: Perovskite
Temperature Sensor/Device Temperature: TC-K, 25.7 ± 1 °C
Environmental conditions at the time of calibration: Temperature: 24 ± 3 °C; Humidity: 40 ± 10 %

The above DUT has been tested using the following methods to meet the ISO 17025 Standard by the PV Lab at Newport Corporation. Quoted uncertainties are expanded using a coverage factor of $k = 2$ and expressed with an approximately 95% level of confidence. Measurement of total irradiance is traceable to the World Radiometric Reference (WRR) and all other measurements and uncertainties are traceable to either NIST or CNRC and the International System of Units (SI). The performance parameters reported in this certificate apply only at the time of the test, and do not imply future performance. Performance parameters were obtained under equilibrium conditions, as described in the **DUT Calibration Procedures** section below.

Efficency [%]	19.77	±	0.63	V_oc [V]	1.1204	±	0.0086	I_sc [A]	0.002362	±	0.000050
P_max [mW]	1.989	±	0.059	V_max [V]	0.9255	±	0.0131	I_max [A]	0.002149	±	0.000049
FF [%]	75.2	±	1.4	Area [cm^2]	0.1006	±	0.0005	M	0.970	±	0.015

Methods:

I-V: ASTM E948-16 *Standard Test Method for Electrical Performance of Photovoltaic Cells Using Reference Cells Under Simulated Sunlight*

QE: ASTM E1021-15 *Standard Test Method for Spectral Responsivity Measurements of Photovoltaic Devices*

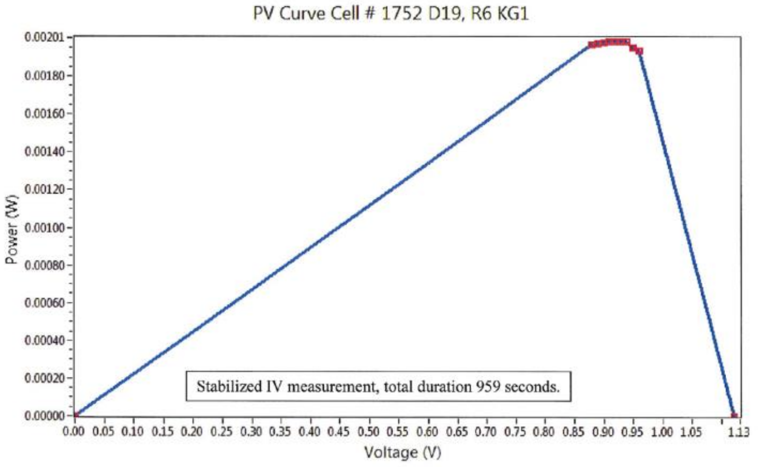

Supplementary Figure 17| Certified stabilized efficiency. Certified stabilized efficiency for a target device. All the parameters are stabilized. A bias voltage-stabilized out power curve is included.

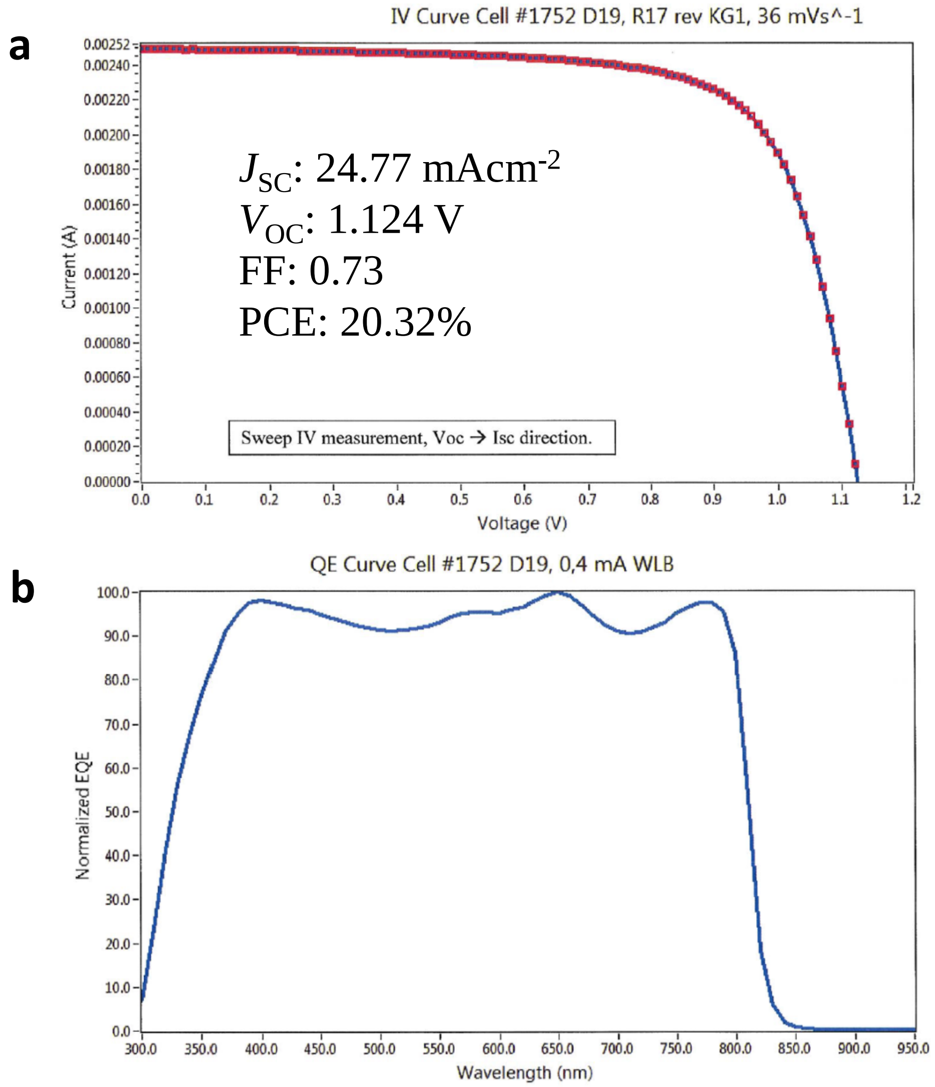

Supplementary Figure 18| *J-V* and EQE curves of the certified device. (**a**) Current density-voltage (*J-V*) curve and (**b**) normalized external quantum efficiency (EQE) curve for the certified device provided by Newport Corporation. The *J-V* curve was obtained from reverse scan (from V_{OC} to J_{SC}) with scan rate of 36 mVs^{-1}.

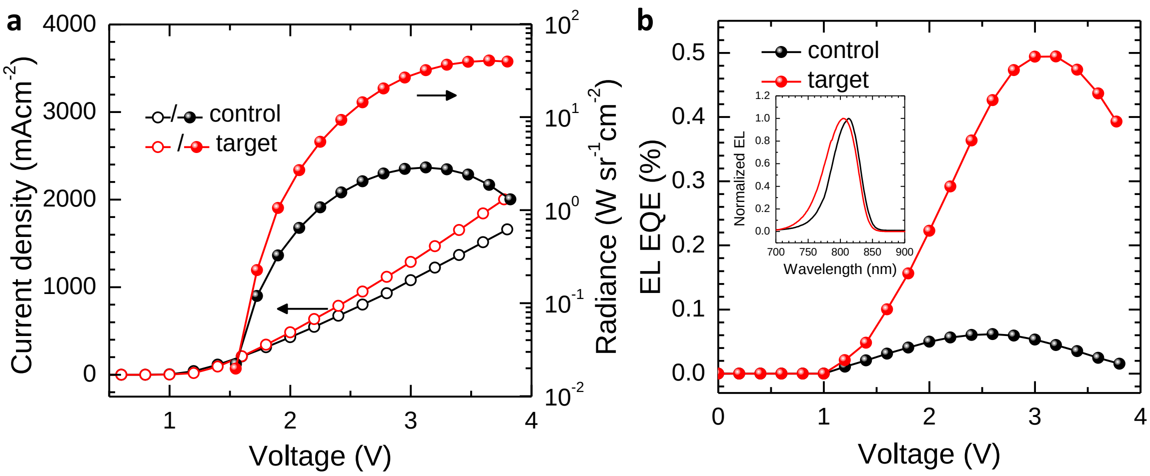

Supplementary Figure 19| Electroluminescence properties of control and target devices. (**a**) Current density-voltage curve and measured radiance of the control and target devices. (**b**) Calculated electroluminescence external quantum efficiency (EL EQE) spectra for the control and target devices. Inset of B shows normalized EL spectra of the devices at bias voltage of 2 V.

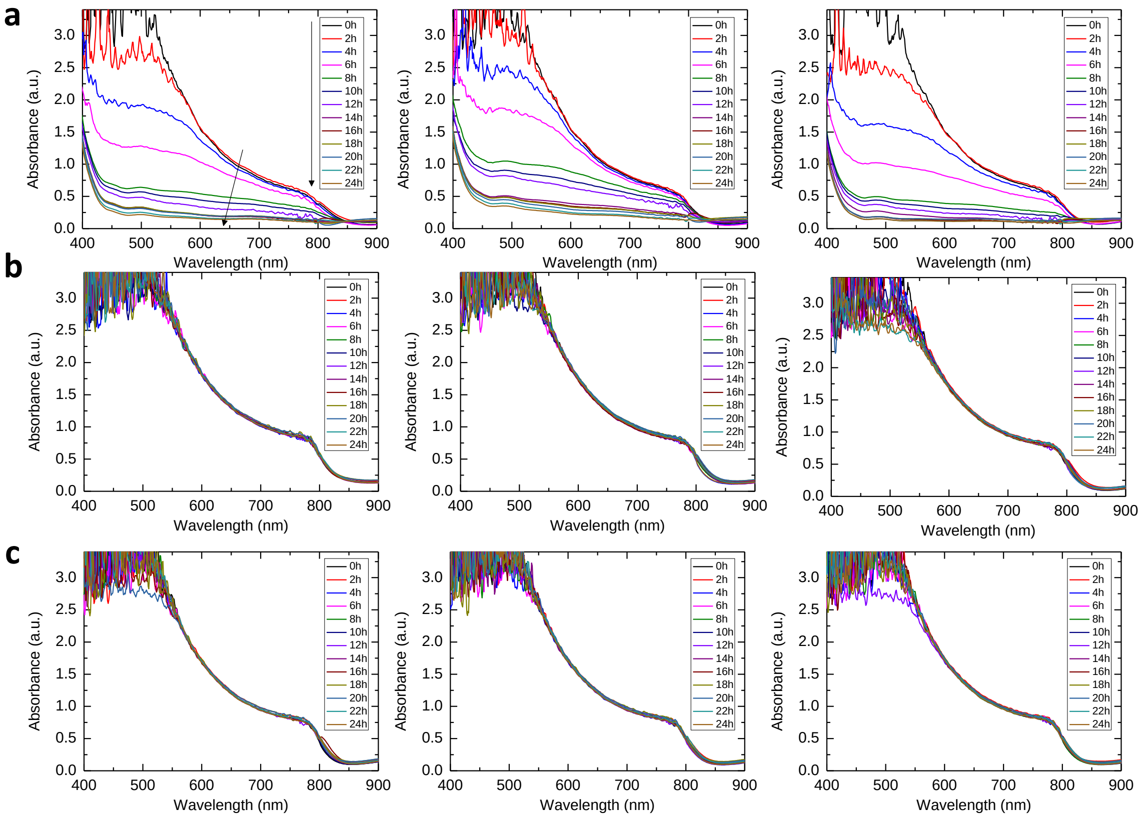

Supplementary Figure 20| Evolution of absorption under high relative humidity (RH). Evolution of absorption spectra under relative humidity of 80±5%.(**a**) bare $FAPbI_3$, (**b**) $FAPbI_3$ with 1.67 mol% PEA_2PbI_4 and (**c**) $FA_{0.98}Cs_{0.2}PbI_3$ with 1.67 mol% PEA_2PbI_4. Three films were tested for each composition.

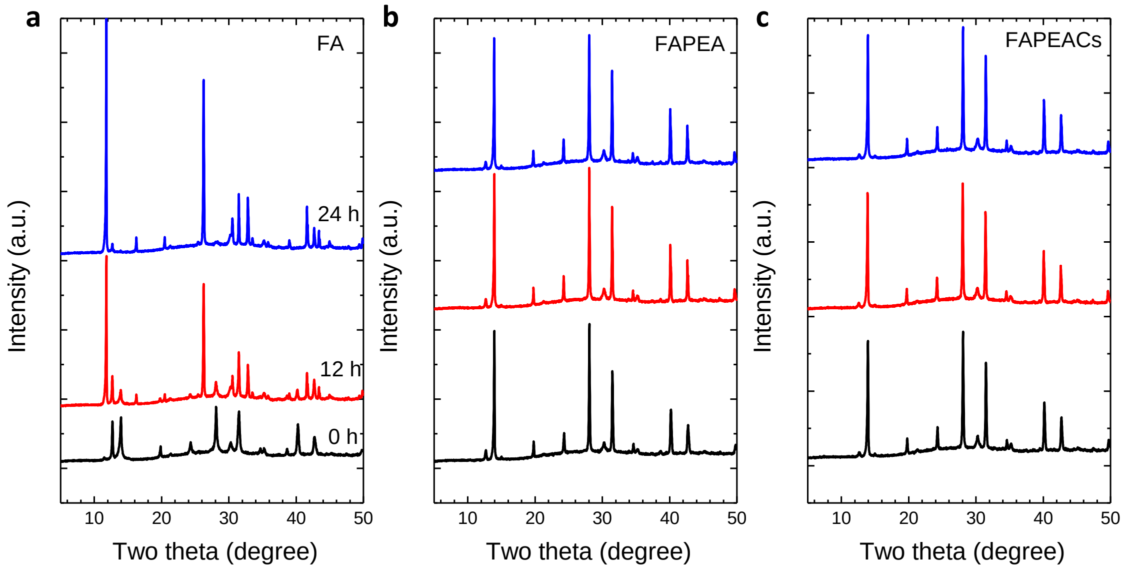

Supplementary Figure 21| Evolution of X-ray diffraction patterns. Evolution of XRD patterns of the perovskite films under relative humidity (RH) of 80±5%. (**a**) bare $FAPbI_3$ (FA), (**b**) $FAPbI_3$ with 1.67 mol% PEA_2PbI_4 (FAPEA) and **c**, $FA_{0.98}Cs_{0.2}PbI_3$ with 1.67 mol% PEA_2PbI_4 (FAPEACs).

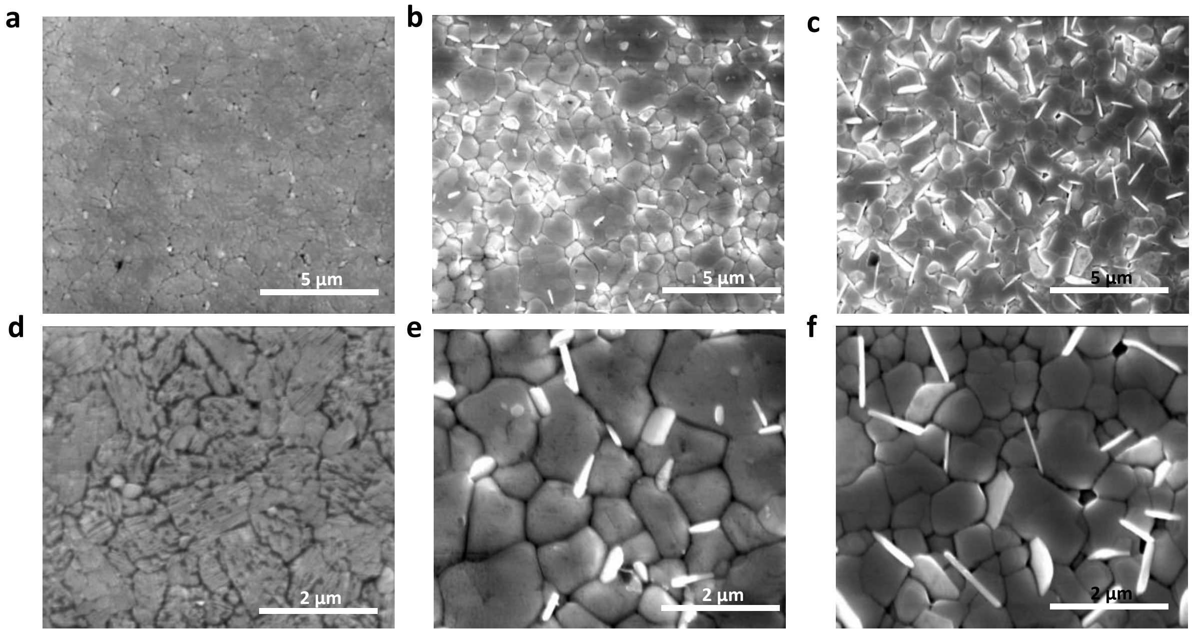

Supplementary Figure 22| Morphology of $FAPbI_3$ film with added 2D perovskite. Surface scanning electron microscopic (SEM) images of (**a**, **d**) bare $FAPbI_3$, (**b, e**) $FAPbI_3$ with 1.67 mol% PEA_2PbI_4 and (**c**, **f**) $FAPbI_3$ with 10.00 mol% PEA_2PbI_4 . All the films were annealed at 150 ^oC for 10 min.

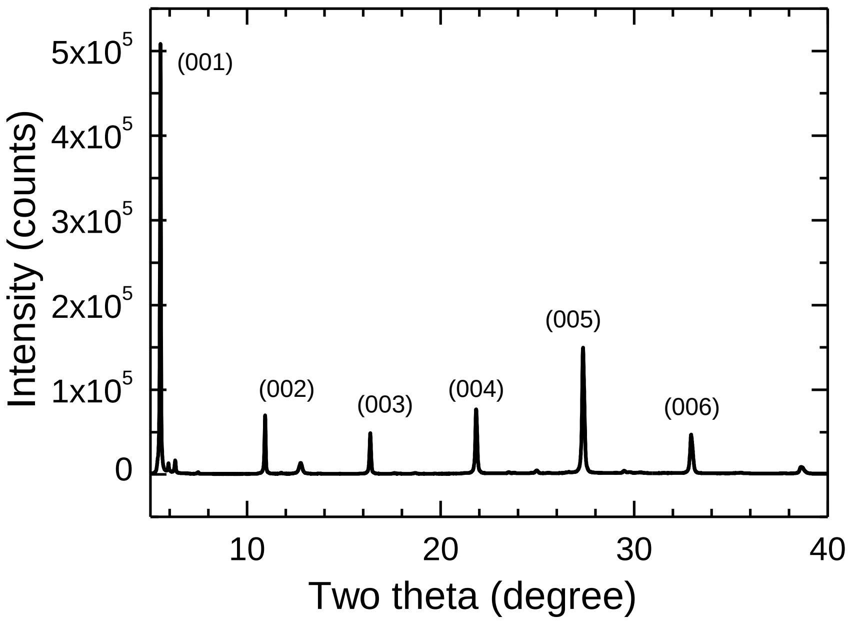

Supplementary Figure 23| X-ray diffraction pattern of the PEA_2PbI_4 perovskite film. The film was coated on ITO substrate.[1]

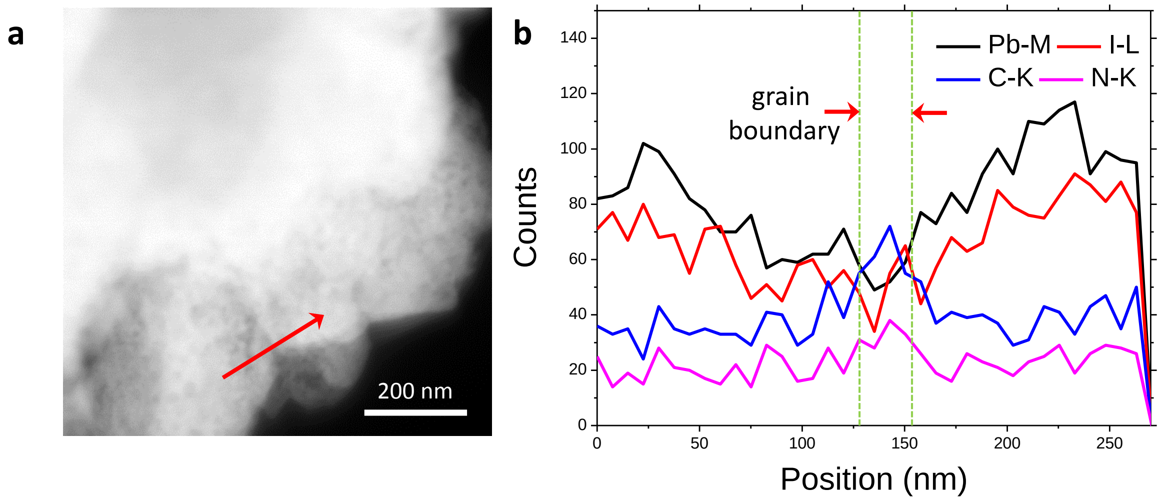

Supplementary Figure 24| Energy dispersive X-ray spectroscopic (EDS) analysis. (**a**) Drift corrected scanning transmission electron microscopic (STEM) image of the $FA_{0.98}Cs_{0.02}PbI_3$ perovskite with 1.67 mol% of PEA_2PbI_4 perovskite. A red arrow indicates the profile where EDS line analysis was performed. (**b**) Elemental distribution profile obtained from the EDS line scan. Grain boundary region is indicated with dashed line and red arrows.

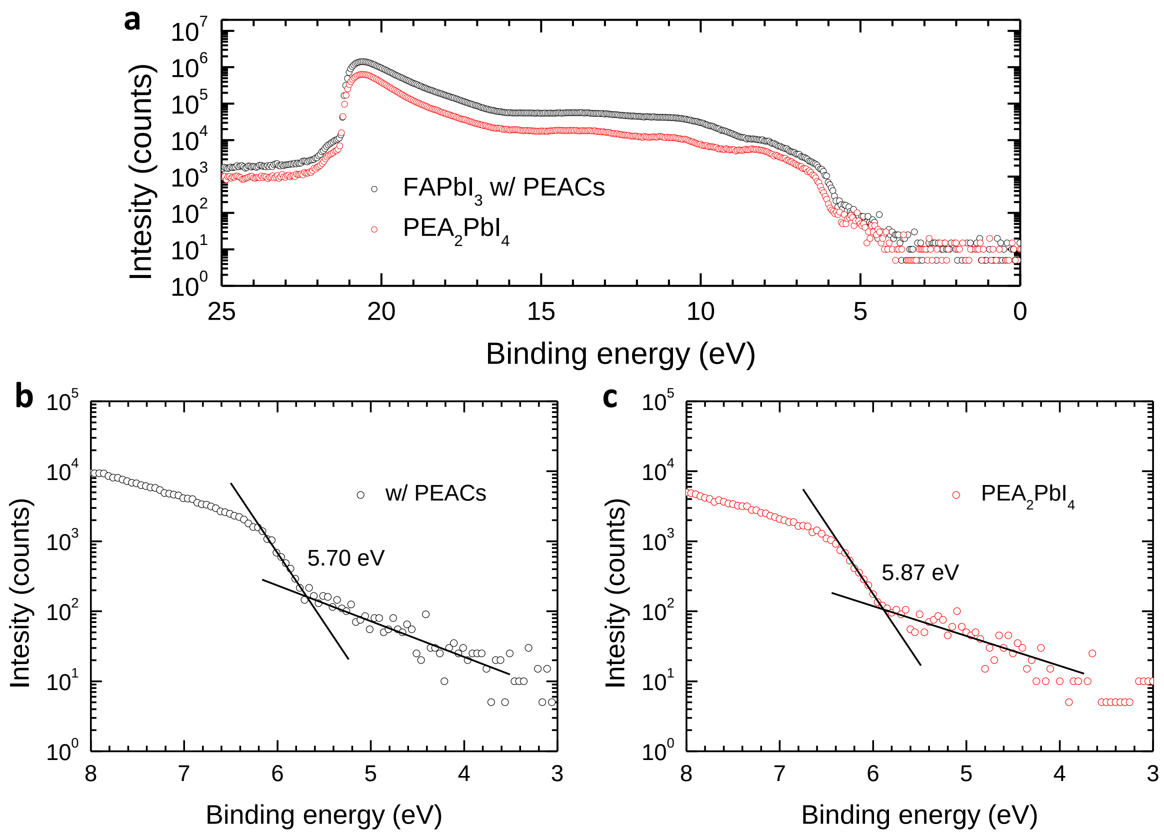

Supplementary Figure 25| Ultraviolet photoelectron spectroscopic (UPS) analysis. UPS analysis of $FA_{0.98}Cs_{0.02}PbI_3$ with 1.67 mol% PEA_2PbI_4 and pure PEA_2PbI_4 perovskite films. (**a**) Complete spectra and low binding energy onset of (**b**) $FA_{0.98}Cs_{0.02}PbI_3$ with 1.67 mol% PEA_2PbI_4 and (**c**) pure PEA_2PbI_4 perovskite films.

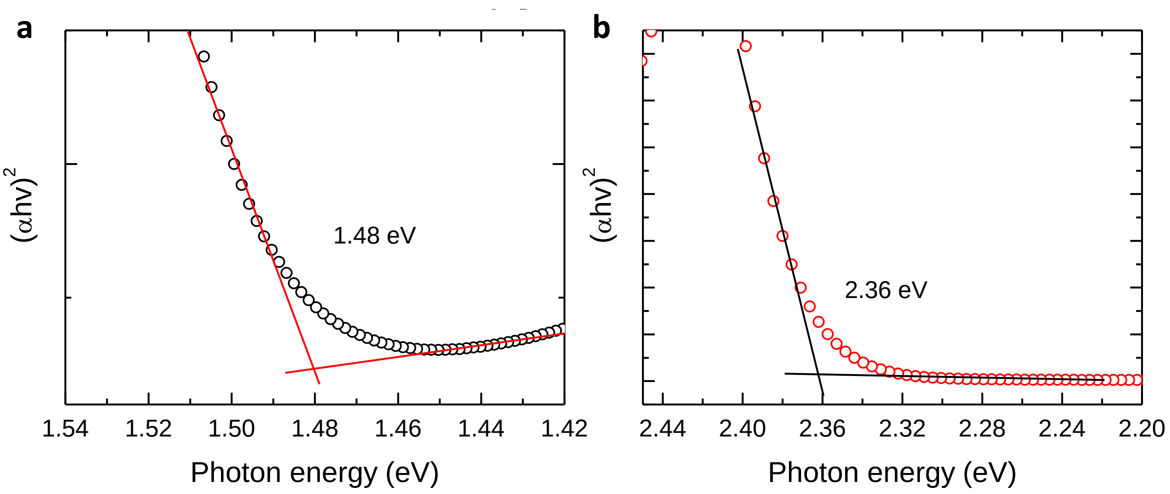

Supplementary Figure 26| Determination of bandgap using a Tauc plot. Tauc plots for (**a**) $FA_{0.98}Cs_{0.02}PbI_3$ perovskite with 1.67 mol% PEA_2PbI_4 and (**b**) pure PEA_2PbI_4 perovskite films. Onset region was fitted to derive the optical bandgap.

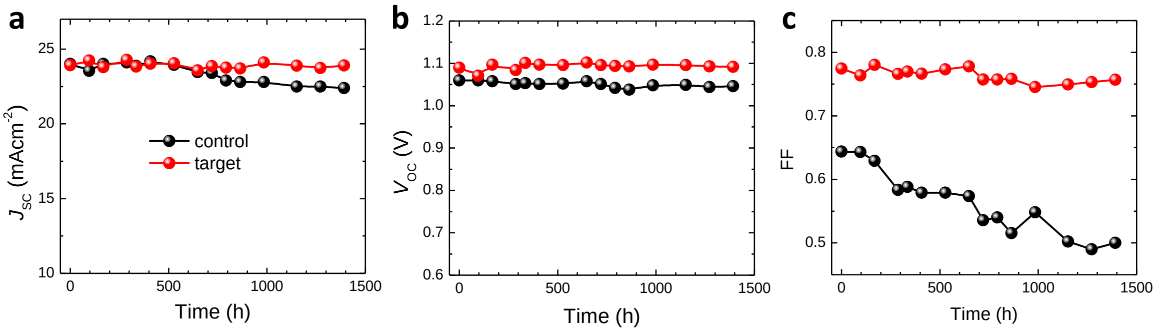

Supplementary Figure 27| Ambient stability test of control and target devices. Evolution of (**a**) short-circuit current density (J_{SC}), (**b**) open circuit voltage (V_{OC}) and (**c**) fill factor (FF) of the control and target device.

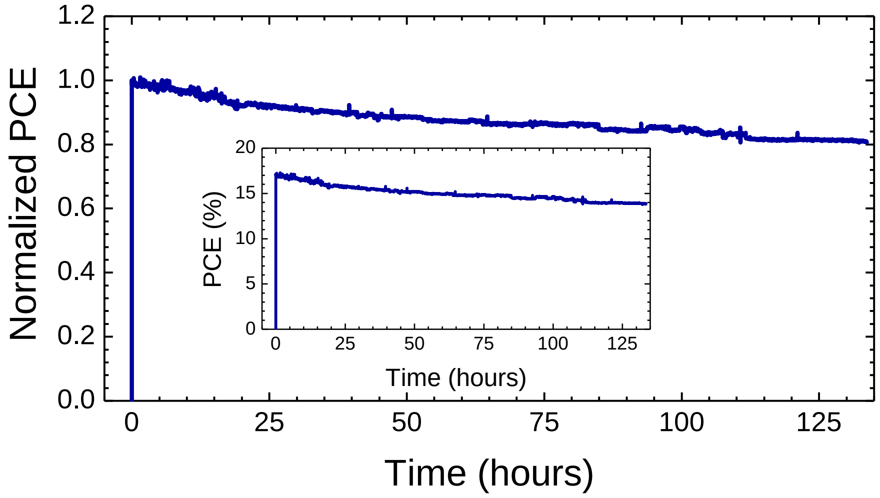

Supplementary Figure 28| Maximum power point tracking of the target device. The measurement was performed under 1 sun illumination in ambient condition with encapsulation. Inset of the figure shows the PCE without normalization.

Supplementary Table 1| Fitted parameters for time resolved photoluminescence deacy. Biexponential and triexponential decay models were used for bare $FAPbI_3$ and $FAPbI_3$ with 2D perovskite respectively. The values in parenthesis indicate proportion of the each decay component.

	$FAPbI_3$	w/ PEA_2PbI_2	w/ PEA_2PbI_4 + Cs
A_1	672.6 (51.8%)	491.2 (46.5%)	446.1 (42.4%)
τ_1	3.0	3.2	3.2
A_2	625.4 (48.2%)	212.8 (20.2%)	177.8 (16.9%)
τ_2	78.5	148.7	177.7
A_3		351.4 (33.3%)	428.2 (40.7%)
τ_3		1037.5	1140.2
	39.4	376.9	495.4

Supplementary Table 2| Improved photovoltaic parameters with 2D perovskite and Cs. Photovoltaic parameters of the device incorporating bare $FAPbI_3$ (FA), $FAPbI_3$ with 1.67 mol% PEA_2PbI_4 (FAPEA) and $FA_{0.98}Cs_{0.02}PbI_3$ with 1.67 mol% PEA_2PbI_4 (FAPEACs). The photovoltaic parameters are obtained from reverse scan (from V_{OC} to J_{SC}) with scan rate of 0.1 Vs^{-1}. All the devices were fabricated in same batch.

	J_{SC} ($mAcm^{-2}$)	V_{OC} (V)	FF	PCE (%)
FA	23.82±0.27	1.023±0.018	0.654±0.012	15.95±0.36
FAPEA	24.16±0.40	1.044±0.018	0.717±0.016	18.08±0.52
FAPEACs	24.37±0.25	1.061±0.014	0.741±0.009	19.16±0.37

Supplementary Table 3| *d* spacing values for $FA_{0.98}Cs_{0.02}PbI_3$ with 1.67 mol% PEA_2PbI_4. The values were calculated from X-ray diffraction pattern in Supplementary Fig. 8 using Bragg`s law.[2]

hkl	*d* (Å)
001	6.35
011	4.49
111	3.67
002	3.18
012	2.84
022	2.25
003	2.12

Supplementary Table 4| Calculated *d* spacing values for PEA_2PbI_4. The values were calculated from X-ray diffraction pattern in Supplementary Fig. 18 using Bragg`s law.[1]

hkl	*d* (Å)
001	16.0
002	8.10
003	5.42
004	4.07
005	3.26
006	2.72

Supplementary Note 1

Optimization of the device by cesium incorporation

For optimization of the device, we first controlled the concentration and heat-treatment process for $FAPbI_3$ with 1.67 mol% PEA_2PbI_4 perovskite. The data in **Supplementary Figs. 2** and **4** was obtained using low concentration (1 mmol of perovskite precursors in 600 mg of DMF) precursor solution with same annealing time (10 min at 150 ^{o}C) to avoid the effect of annealing time on crystallinity. With 2D perovskite, we were able to prolong the annealing time since it was thermally more stable compared to bare $FAPbI_3$. The optimized annealing time was 20 min at 150 ^{o}C with 2D perovskite (For bare $FAPbI_3$, the optimum annealing time was 10 min). Optimization of the heat-treatment and concentration (1 mmol of perovskite precursors in 560 mg of DMF) enhanced the average PCE up to 17.86±0.56% (**Supplementary Fig. 8**, with 0 mol% Cs). In addition to this, we noticed that incorporation of small amount of Cs (2 mol%) further improve the average PCE up to 19.00±0.69% due to enhancement in V_{OC} and FF (**Supplementary Fig. S8**). The origin of the improvement might be passivation of defects as we discussed in the main text (bandgap of the perovskite was not changed, see **Supplementary Note 2**).

Supplementary Note 2

X-ray diffraction and absorption properties with 2 mol% Cs

It has been reported that incorporation of relatively smaller Cs cation into $FAPbI_3$ can increase the bandgap of the perovskite.[3,4] In the previous studies, typically higher than 5 mol% of the Cs was used to stabilize the $FAPbI_3$ while we incorporated only 2 mol% of Cs. We compare the X-ray diffraction (XRD) pattern and absorption spectrum of the perovskite films without and with 2 mol% Cs. As seen in **Supplementary Fig. S9a**, XRD patterns were similar with and without 2 mol% Cs. The absorption spectra of the films with and without 2 mol% Cs were also almost identical as seen in **Supplementary Fig. 9b,** which indicates bandgap of the perovskite is maintained after addition of 2 mol% of Cs (The bandgap was determined to be 1.48 eV as seen in **Supplementary Fig. 26**). As a result, external quantum efficiency (EQE) spectrum of our target device shows almost identical onset (rather slightly red shifted) with bare $FAPbI_3$ device (**Fig. 2d** in main text). Surface scanning electron microscopic (SEM) images of the films were compared in **Supplementary Fig. 10**. Relatively larger grains without specific shape were observed with bare $FAPbI_3$ while relatively smaller granular grains were observed with 2D perovskite. The grain size was decreased with 2 mol% of Cs, which is correlated with slight decrease of peak intensity in XRD spectrum.

Supplementary Note 3

Impact of 2 mol% Cs on stability and optoelectronic properties of $FAPbI_3$

Typically, at least 10 mol% replacement of FA with Cs (5 mol% in combination with 17 mol% of MA) is required to stabilize the black perovskite phase in case of pure $FAPbI_3$.[3-5] We believe that the 2 mol% replacement of FA with Cs reduces the defect density as observed from time-resolved photoluminescence in **Fig. 2**, but it do not play critical roles in our system. For example, we compared phase-stability of the $FAPbI_3$ and $FA_{0.98}Cs_{0.02}PbI_3$ perovskite film under relative humidity of 70±5% (**Supplementary Fig. 11**). As can be seen in the photos and X-ray diffraction (XRD) patterns of the films, both of $FAPbI_3$ and $FA_{0.98}Cs_{0.02}PbI_3$ perovskite films were rapidly degrade within 24 h. The black color of the films were rapidly changed to yellow, which can be attributed to conversion of the cubic perovskite phase to yellow hexagonal non-perovskite phase. We also compared the steady-state and time-resolved photoluminescence (PL) properties of the $FAPbI_3$ and $FA_{0.98}Cs_{0.02}PbI_3$ perovskite films in **Supplementary Fig. 12**. As seen in **Supplementary Fig. 12a**, the peak PL intensity of bare $FA_{0.98}Cs_{0.02}PbI_3$ is $9.39x10^5$ which was improved to 1.17 $x10^6$ with 2 mol% of Cs. The PL lifetime of $FAPbI_3$ (40.4 ns) was prolonged to 52.0 ns with incorporation of 2 mol% Cs. However, the improvement in PL intensity and lifetime is much less compared to that observed with addition of 2D perovskite (around 5 and 10 times enhancement in PL intensity and lifetime, respectively). Therefore, we concluded that improvement of the phase-stability and optoelectronic quality of the perovskite film is predominantly due to the addition of 2D perovskite.

Supplementary Note 4

Presence of 2D perovskite

In **Supplementary Figs. 10b** and **c**, bright colored plates are sparsely observed with addition of 1.67 mol% of 2D perovskite into precursor solution. The morphology of plates in **Supplementary Figs. 10b** and **c** is clearly distinguished from one in **Supplementary Fig. 10a**, which might be the thermally induced PbI_2. We speculated the plates are crystallized 2D perovskite. Although it was not detected in XRD patterns, surface SEM images in **Supplementary Fig. 22** support this speculation. Compared to bare $FAPbI_3$ (**Supplementary Figs. 22a** and **d**), the bright colored plates were formed between grains with addition of 5 mol% of 2D perovskite (**Supplementary Figs. 22b** and **e**). The amount and size of the plates were further enhanced with addition of 10 mol% of 2D perovskite (**Supplementary Figs. 22c** and **f**) although the amount of PbI_2 in XRD was not increased (**Supplementary Fig. 4**).

Supplementary References

1. Ma, D. *et al.* Single-crystal microplates of two-dimensional organic–inorganic lead halide layered perovskites for optoelectronics. *Nano Research* **10**, 2117-2129 (2017).
2. Saidaminov, M. I., Abdelhady, A. L., Maculan, G. & Bakr, O. M. Retrograde solubility of formamidinium and methylammonium lead halide perovskites enabling rapid single crystal growth. *Chem. Commun.* **51**, 17658-17661 (2015).
3. Lee, J. W. *et al.* Formamidinium and Cesium Hybridization for Photo-and Moisture-Stable Perovskite Solar Cell. *Adv. Energy Mater.* **5**, 1501310 (2015).
4. Saliba, M. *et al.* Cesium-containing triple cation perovskite solar cells: improved stability, reproducibility and high efficiency. *Energy Environ. Sci.* **9**, 1989-1997 (2016).
5. McMeekin, D. P. *et al.* A mixed-cation lead mixed-halide perovskite absorber for tandem solar cells. *Science* **351**, 151-155 (2016).
